# The Knowledge Assessment of Cardiovascular Disease Risk Factors: A Cross-Sectional Study

**DOI:** 10.7759/cureus.59774

**Published:** 2024-05-07

**Authors:** Nora Taiek, Nour El Houda El Fadili, Abderrahmane Belkacem, Attoumane Abdou Cheikh, Kaoutar Kabbadj, Narjisse Damoun, Faiza Aziouaz, Abdelkader Jalil El Hangouche

**Affiliations:** 1 Department of Physiology, Faculty of Medicine and Pharmacy of Tangier, Abdelmalek Essaadi University, Tangier, MAR

**Keywords:** awareness of cardiovascular disease, cardiac risk factors and prevention, level of knowledge, cardiovascular diseases, cardiovascular disease risk factor

## Abstract

Introduction: Cardiovascular diseases (CVDs) are the primary cause of mortality worldwide. Numerous factors can indicate the likelihood of developing CVDs. Gaining a comprehensive understanding of these risk factors is the initial step towards implementing successful preventive measures to defy the prevalence of CVDs across all demographics. The aim of this study is to evaluate the Moroccan population's level of knowledge regarding cardiovascular risk factors (CVRF).

Methods: This questionnaire-based cross-sectional study was conducted among 744 participants. Their knowledge of CVD risk factors was assessed by the Heart Disease Facts Questionnaire (HDFQ). Socio-demographic characteristics were collected and statistical analyses were performed using Statistical Package for the Social Sciences (IBM SPSS Statistics for Windows, IBM Corp., Version 26.0, Armonk, NY).

Results: Among 744 participants, 475 (63%) were male and 409 (55%) were young adults. The mean HDFQ score was 64.36%. Overall, 47.4% of the respondents were aware of CVD risk factors, 27% had moderate knowledge and 25.6% had poor knowledge. The most commonly identified factors were smoking (86.8%), obesity (85.6%), and aging (80.5%). Age was the only factor that showed a significant association with the awareness of CVD risk factors.

Conclusion: The level of knowledge of CVRF is moderate among the Moroccan population. Effective health education about CVRF and adequate prevention measures is certainly essential to minimize the burden of CVD.

## Introduction

The rising burden of cardiovascular diseases (CVDs) has become a major public health problem worldwide [1]. CVDs are a group of diseases that predominantly impact the heart and blood vessels [1]. These diseases are usually associated with atherosclerosis and an elevated risk of thrombosis due to blood clots [2]. CVDs include diseases such as coronary heart diseases, peripheral vascular diseases, congenital heart diseases, pulmonary embolism, cerebrovascular diseases, and venous thrombosis [1].

Cardiovascular risk factors (CVRFs) have an important role in the development of almost all CVDs [3]. They can be divided into two categories: modifiable factors, including hypertension, diabetes, high cholesterol, obesity, physical inactivity, and inadequate intake of fruits and vegetables [4]; and non-modifiable factors such as age, sex [4], family history and ethnicity [5,6].

CVDs remain the principal cause of death globally, with an estimation of 17.9 million deaths (32% of global deaths) each year reaching 23.3 million deaths by 2030, 85% of deaths are due to stroke and heart disease, and one-third of these fatalities are premature in adults under the age of 70 [1]. As reported in various studies, the prevalence of these risk factors is on the rise across nearly all regions of Africa [7]. According to the World Health Organization (WHO), “over three-quarters of CVD deaths take place in low- and middle-income countries” [1].

The Centers for Disease Control and Prevention report that a significant portion of deaths (six out of 10 deaths) caused by CVDs can be prevented [8]. Previous studies have demonstrated that increasing knowledge about CVDs can effectively reduce an individual's actual risk of developing the disease [9]. Hence, it is important to possess knowledge regarding the implications, signs, and potential factors that are linked to CVDs [10].

A limited number of studies have addressed CVDs’ level of knowledge worldwide [11]. The reason why this study was conducted is to determine the level of knowledge regarding CVDs in the general population of Morocco.

## Materials and methods

Type and date of the study

A cross-sectional descriptive survey was carried out from May to August 2022 among the Moroccan population.

Sample size

**Figure 1:**
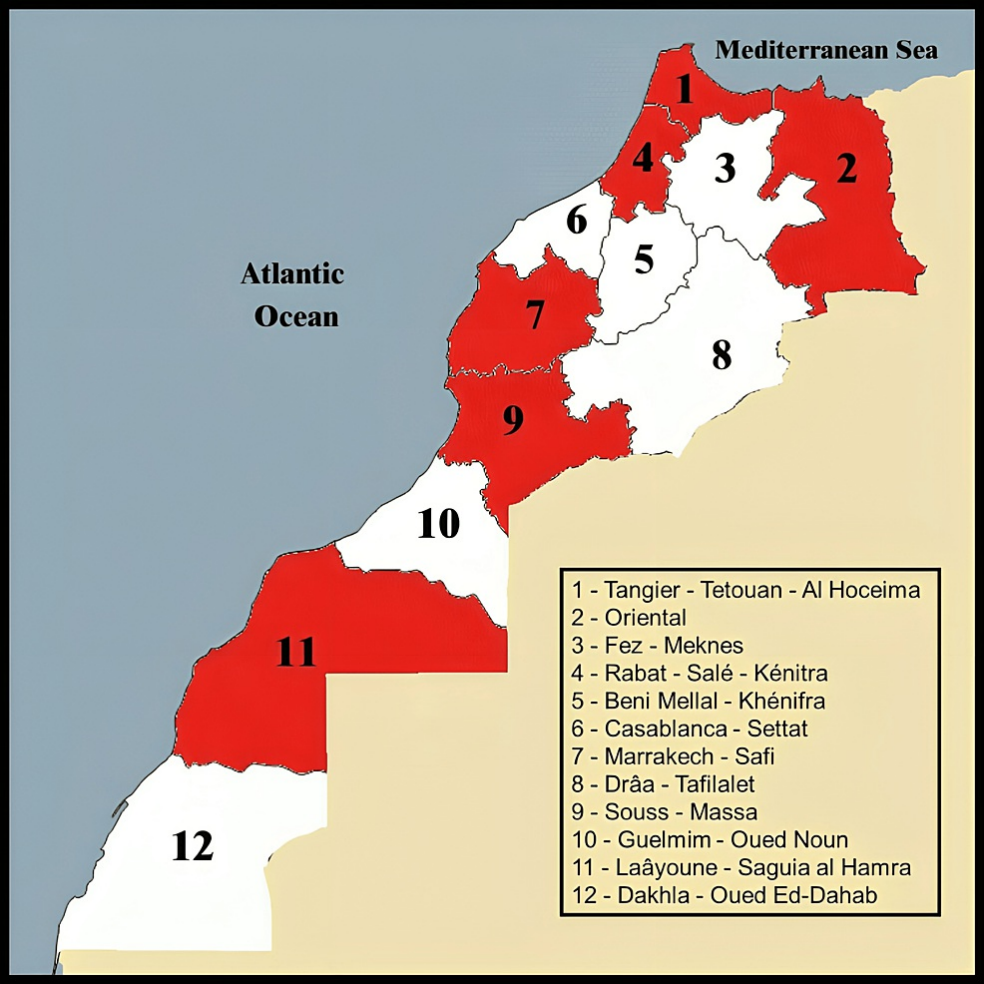
Geographical distribution of regions included in the study The image is created by the authors.

An estimated sample size of 385 was calculated using a margin of error of 5% and a confidence level of 95% [12]. A total of 744 adults aged more than 16 years were recruited from public places in various Moroccan cities (Figure 1).

This study was randomly carried out every day from 9h00 to 18h00 in six regions of Morocco, in each region two recruitment sites were placed; one in a rural area and another in an urban area. A number of 62 participants were recruited from the weekly markets in villages while 62 participants were from downtown.

Data collection and instrument

Individuals who consented to participate in this study were interviewed through a pretested standard self-administered questionnaire after obtaining written informed consent. The questionnaire consisted of three sections assessing the following items; The first section includes written consent of the participants, the sentence “Response to this questionnaire is voluntary and anonymous. The data collected will be used for scientific research purposes only. Do you give your consent to participate in this study?” was added at the top of the questionnaire form. The second section records demographic indicators (age, gender, marital status, academic level, profession) and tobacco consumption. The last section assesses the level of knowledge of CVRFs using the French-validated version of the 25-item Heart Disease Fact Questionnaire (HDFQ) [13] originally designed by Wagner et al. [14].

Participants were asked to respond to each statement by choosing a single option: "True", "False", or "I don’t know" [15]. Each correct response was scored “one” and each incorrect response or “I don’t know” was scored “zero”.

The total score ranges from 0% to 100%; it is calculated by multiplying the number of correct responses by four. A low level of knowledge is defined with a score of <50%, a moderate level of knowledge is defined with a score between 50% and 70% while a good level of knowledge includes the participants with an HDFQ score >70% [16].

The data were collected and transposed into an Excel spreadsheet (Microsoft® Corp., Redmond, WA), then analyzed using the Statistical Package for the Social Sciences (IBM SPSS Statistics for Windows, IBM Corp., Version 26.0, Armonk, NY). The categorical variables were expressed in numerical form, along with their corresponding percentages.

To determine the factors linked to the level of knowledge, both univariate and multivariate logistic regression models were performed. P < 0.05 was taken as statistically significant.

## Results

This study involved 744 participants, with the predominance of the female gender (63%). Participants' ages range from 16 to 84 years old. The categorical distributions for young adults, middle-aged adults, and old-aged adults were 55%, 30.6%, and 14.4%, respectively. In this study, 51.7% of the participants were single, while 41.9% were married. In terms of the participants' educational background, 58.1% were university graduates, and only 8.2% were illiterate. Regarding tobacco consumption, the majority of the participants (86.7%) were non-smokers, 9.9% were smokers, and only 3.4% were ex-smokers. The rest of the socio-demographic characteristics are displayed in Table 1.

**Table 1:** Socio-demographic characteristics of the study population (n=744) CVD: cardiovascular disease

Characteristics	n (%)	95% Confidence Interval	
		Inf	Sup
Age			
Young adults (16-30)	409 (55.0)	51.3	58.5
Middle-aged (31-45)	228 (30.6)	27.2	34.1
Old-aged adults (>45)	107 (14.4)	11.8	17.1
Gender			
Female	475 (63.8)	60.3	67.2
Male	269 (36.2)	32.8	39.7
Marital status			
Single	385 (51.7)	48.4	55.5
Married	312 (41.9)	38.0	45.4
Divorced	26 (3.5)	2.2	5.0
Widowed	21 (2.8)	1.7	4.2
Educational level			
Illiterate	61 (8.2)	6.3	10.2
Primary	91 (12.2)	9.9	14.8
Secondary	160 (21.5)	18.8	24.6
University degree	432 (58.1)	54.4	61.6
Profession			
Student	216 (29.0)	25.9	32.5
Employed	193 (25.9)	22.6	29.0
Liberal profession	156 (21.0)	18.0	23.9
Retired	11 (1.5)	0.7	2.4
Unemployed	168 (22.6)	19.8	25.5
Smoking status			
Non-smoker	645 (86.7)	84.0	89.1
Smoker	74 (9.9)	7.8	12.1
Ex-smoker	25 (3.4)	2.2	4.8
Family medical history of CVD			
Yes	485 (65.2)	61.6	68.4
No	212 (28.5)	25.3	31.9
I don’t know	47 (6.3)	4.7	8.1
Personal medical history of CVD			
Yes	147 (19.8)	16.8	22.8
No	597 (80.2)	77.2	83.2

Concerning the 25 statements of the HDFQ, 10 questions were scored above 70%, 11 questions were scored between 50% and 70%, and four questions were scored below 50%.

The mean knowledge total score was 64.36 ± 21.86 of which 47.4% have good knowledge (score over 70%) while 27% have moderate knowledge (score between 50 and 70%) and 25.6% have low knowledge (score less than 50%).

Participants showed a solid knowledge of certain CVRFs, such as smoking (86.8%), being overweight (85.6%), aging (80.5%), high blood pressure (75.3%), and eating fatty food (73.9%).

The majority also demonstrated adequate knowledge regarding several CVD prevention measures, such as regular physical activity (87.2%) and smoking cessation (78.5%). However, blood pressure control and diabetes were recognized by 68.7% and 64.5% of the participants, respectively.

In contrast, low high-density lipoprotein (HDL) cholesterol (35.6%) was not considered a CVRF. Also, 34.1% of the participants were not aware that people with diabetes are more likely to have high cholesterol and only 25% were able to know the relationship between diabetes and the tendency to have low HDL cholesterol. The participants' knowledge regarding CVRFs is displayed in Table 2.

**Table 2:** Participants’ knowledge regarding cardiovascular risk factors (n=744)

Nº	Item	Correct answer [13]	Correct (%)	Incorrect (%)
1.	A person always knows when they have heart disease	False	434 (58.3)	310 (41.6)
2.	If you have a family history of heart disease, you are at risk for developing heart disease	True	426 (57.3)	312 (42.7)
3	The older a person is, the greater their risk of developing heart disease	True	599 (80.5)	145 (19.5)
4	Smoking is a risk factor for heart disease	True	646 (86.8)	98 (13.2)
5	A person who stops smoking will lower their risk of developing heart disease	True	584 (78.5)	160 (21.5)
6	High blood pressure is a risk factor for heart disease	True	560 (75.3)	184(24.7)
7	Keeping blood pressure under control will reduce a person’s risk for developing heart disease	True	511 (68.7)	233 (31.3)
8	High cholesterol is a risk factor for developing heart disease	True	543 (73)	201 (27)
9	Eating fatty foods does not affect blood cholesterol levels	False	550 (73.9)	194 (26.1)
10	If your “good” cholesterol (HDL) is high, you are at risk for heart disease	False	265 (35.6)	479 (64.4)
11	If your “bad” cholesterol (LDL) is high, you are at risk for heart disease	True	440 (59.1)	304 (40.9)
12	Being overweight increases a person’s risk for heart disease	True	637 (85.6)	107 (14.4)
13	Regular physical activity will lower a person’s chance of getting heart disease	True	649 (87.2)	95 (12.8)
14	Only exercising at a gym or in an exercise class will lower a person’s chance of developing heart disease	False	542 (72.8)	202 (27.2)
15	Walking and gardening are considered an exercise that will help lower a person’s chance of developing heart disease	True	616 (82.8)	128 (17.2)
16	Diabetes is a risk factor for developing heart disease	True	480 (64.5)	264 (35.5)
17	High blood sugar puts a strain on the heart	True	485 (65.2)	259 (34.8)
18	If your blood sugar is high over several months, it can cause your cholesterol level to go up and increase the risk of heart disease	True	433 (58.2)	311 (41.8)
19	A person who has diabetes can reduce their risk of developing heart disease if they keep their blood sugar levels under control	True	475 (63.8)	269 (36.2)
20	People with diabetes rarely have high cholesterol	False	254 (34.1)	490 (65.9)
21	If a person has diabetes keeping their cholesterol under control will help to lower their chance of developing heart disease	True	454 (61)	290 (39)
22	People with diabetes tend to have low HDL cholesterol	True	186 (25)	558 (75)
23	A person who has diabetes can reduce their risk of developing heart disease if they keep their blood pressure under control	True	450 (60.5)	294 (39.5)
24	A person who has diabetes can reduce their risk of developing heart disease if they keep their weight under control	True	494 (66.4)	250 (33.6)
25	Men with diabetes have a higher risk of heart disease than women with diabetes	False	258 (34.7)	486 (65.3)

Statistical analysis disclosed a statistically significant association between the level of knowledge and age, marital status, profession, smoking status, prior knowledge of CVRFs, personal history of CVRFs, and family medical history. Whereas, no difference statistically significant in terms of the level of knowledge of CVRFs between groups of different educational levels and gender was found (Table 3).

**Table 3:** Relationship between total score of cardiovascular risk factors knowledge with demographic and health-related variables (n=744) Bold values indicate statistical significance at p<0.05. CVRFs: cardiovascular risk factors p-values derived from the Mann-Whitney U test and Kruskal-Wallis-Test.

Variable	Median (IQR)	95% Confidence Interval		p-value
		Inf	Sup	
Age				<0.001
Young adults	64 (48-80)	60	68	
Middle-aged adults	76 (52-84)	72	76	
Old-aged adults	76 (52-84)	68	80	
Gender				0.242
Male	68 (44-82)	62.05	72	
Female	68 (52-84)	64	72	
Marital status				<0.001
Single	64 (44-80)	60	65.94	
Married	72 (56-84)	72	76	
Divorced	60 (46-84)	48	80	
Widow	72 (50-78)	60	76	
Education				0.471
Illiterate	76 (50-84)	68	76	
Primary	72 (52-84)	60	76	
Secondary	68 (52-84)	64	74	
University	64 (64-80)	64	68	
Profession				<0.001
Student	62 (44-80)	60	68	
Employed	68 (48-84)	64	76	
Liberal profession	64 (44-80)	60	68	
Retired	84 (80-92)	80	92	
Unemployed	76 (57-84)	72	78	
Prior knowledge of CVRFs				<0.001
Yes	72 (52-84)	68	72	
No	60 (44-80)	56	64	
Personal history of the CVRFs				0.001
Yes	76 (56-84)	68	80	
No	64 (48-80)	64	68	
Smoking status				0.026
Ex-smoker	76 (62-92)	68	91.95	
Smoker	64 (43-80)	56	74	
Non-smoker	68 (48-84)	64	72	
Family medical history				<0.001
Yes	72 (56-84)	68	72	
No	64 (40-80)	56	72	
I don’t know	48 (32-68)	40	56	

Logistic regression models to evaluate the association between participants’ demographic characteristics and CVD awareness are presented in Table 4. The analyses of univariate regression showed that being old-aged (>45 years) (odds ratio (OR) = 2.08; P = <0.001); holding a university degree (OR = 1.927; P = 0.019), being a student (OR = 1.586; P = 0.022), divorced (OR = 0,559; P = <0.001), ex-smoker (OR = 2.413; P = 0.043), and having family medical history (OR = 0.621; P = 0.002) were significantly associated with CVRFs’ level of knowledge. This indicates that old-aged participants have 2.08 times more knowledge than young adults, university degree participants have 1.927 times more knowledge than those with less educational level, and participants with a family medical history showed 37.9% less knowledge compared to those without a family medical history. In the multivariate logistic regression, the only factor that showed a significant association with knowledge of CVRFs was age (OR =2.122; P = 0.002), indicating that participants above 45 years have 2.08 times more knowledge than young adults.

**Table 4:** Results of multi-regression analysis of the association between CVD awareness and sociodemographic (n=744) Variables with a p<0.2 in the univariate analysis were considered for the multivariable analysis. Bold values indicate statistical significance at p<0.05. * refers to the variable used as a reference in the univariate and multivariate regression analysis. CVD: cardiovascular disease

Characteristics	Good awareness	Moderate/poor awareness	Binary regression analysis				Multi-regression analysis				
			P	Odds ratio	Lower CI	Upper CI	P	Odds ratio	Lower CI	Upper CI	
											*Age*											
Young Adults*	161	248		1				1			
Middle-aged Adults	131	97	0.939	1.018	0.64	1.62	0.617	1.148	0.669	1.967	
Old-aged Adults	61	46	<0.001	2.08	1.497	2.891	0.002	2.122	1.312	3.431	
Gender											
Male*	128	141		1				1			
Female	225	250	0.955	0.991	0.735	1.338	0.731	0.934	0.634	1.376	
Educational level											
Illiterate*	37	24		1				1			
Primary	46	45	0.115	1.621	0.889	2.953	0.761	1.114	0.557	2.227	
Secondary	78	82	0.221	1.508	0.781	2.912	0.521	1.26	0.622	2.552	
University degree	192	240	0.019	1.927	1.114	3.332	0.735	1.132	0.553	2.317	
Profession											
Unemployed*	99	69		1				1			
Student	83	133	0.022	1.586	1.07	2.35	0.753	1.079	0.672	1.733	
Employed	96	97	0.167	1.35	0.882	2.064	0.147	1.443	0.88	2.368	
Liberal profession	66	90	0.057	0.22	0.046	1.044	0.119	0.268	0.051	1.404	
Retired	9	2	0.081	0.69	0.454	1.047	0.084	0.62	0.361	1.066	
Marital status											
Single*	158	227		1				1			
Married	173	139	0.796	1.114	0.493	2.518	0.14	2.026	0.794	5.174	
Divorced	10	16	<0.001	0.559	0.414	0.756	0.453	1.195	0.751	1.902	
Widowed	12	9	0.151	0.522	0.215	1.268	0.449	1.52	0.514	4.495	
Tobacco consumption											
Non-smoker*	302	343		1				1			
Smoker	34	40	0.06	2.5	0.96	6.507	0.196	1.933	0.712	5.248	
Ex-smoker	17	8	0.043	2.413	1.027	5.672	0.236	1.75	0.694	4.414	
Family medical history											
No*	103	156		1				1			
Yes	250	235	0.002	0.621	0.457	0.843	0.267	0,823	0.583	1.161	
Personal Medical history of CVD											
No*	247	323		1				1			
Yes	79	68	0.089	0.73	0.508	1.049	0.963	1.01	0.671	1.518	

## Discussion

To the best knowledge of the authors, the assessment of the level of knowledge of CVDs using the HDFQ instrument is conducted for the first time in Morocco. The major outcome of this study revealed that, out of 744 participants, just under half had good awareness of CVRFs and only two participants could correctly identify all the risk factors.

In our study, age, smoking status, marital status, profession, prior knowledge of CVRFs, and family medical history were significantly associated with the total score of CVRF knowledge. On the HDFQ scale, our study highlighted a gap in Moroccan young adults’ CVRF knowledge, knowing that among young adults, 60.6% have an inadequate level of knowledge.

Regarding risk factors, eight out of 10 of the study participants were aware that physical inactivity, smoking, and obesity are risk factors for CVDs, whereas low HDL cholesterol and diabetes were less frequently identified as risk factors [17]. Participants’ awareness about CVRFs was significantly greater among females compared to males, these results are in concordance with former studies conducted in Kuwait [18] and Iran [19], contrary to a study conducted in Saudi Arabia [20].

However, their educational level was not significantly associated with the level of knowledge of CVRFs which was compatible with a Nigerian study [3]. In opposition, participants with a higher educational level were more likely to have a higher level of knowledge in Saudi Arabia [20] and Spain [16].

Our findings stated smoking as the most well-known CVRF in accordance with studies conducted in North America, the Middle East, and South Asian countries [18,21-24]. Smoking cessation was also known by most participants as an indispensable aspect of CVD prevention, as mentioned in Ali S. Alghamdi et al. study [10].

Participants' knowledge about the relationship between controlling blood pressure and reducing the chances of developing heart disease was at best moderate, although most participants were aware that hypertension is a CVRF. These findings were supported by a similar study conducted in Nigeria [3].

The majority of the study participants were also aware that constant physical activity will reduce a person’s chance of getting heart disease, as well as activities such as walking and gardening. Furthermore, our study showed a higher knowledge related to regular physical activity, compared to the figures in Nigeria [3].

The final aspect of the HDFQ reflects the level of knowledge of diabetes and its relationship with heart disease. In our study, this level was averagely moderate. In comparison with other studies, our findings are similar to a study conducted in Oman [21]. Moreover, it was higher in Nigeria [22] and lower in Indonesia [4].

It is known that modifiable risk factors are responsible for more than 90% of the likelihood of developing CVDs. The burden of CVDs can be considerably diminished by emphasizing preventive measures and adopting healthy lifestyle behaviors [23]. As shown in numerous studies, physical activity has proved its efficacy in decreasing the risk of developing CVD by maintaining good physical functioning and improving the quality of life [23,24].

Dietary habits are also considered an important healthy behavior, as expanding confirmations reinforce their powerful modulatory effects on health status and CVRF [25].

Additionally, poor sleep is under-recognized as a CVRF [26]. Numerous studies offer valuable insights into the correlation between the duration of sleep, cardiometabolic risk factors, and significant cardiovascular outcomes [27]. Another study found a U-shaped relationship between sleep duration and CVD outcomes [23]. Therefore, most guidelines recommend seven to eight hours of sleep per night for optimal results [23].

This research highlights the importance of raising awareness of CVRFs among the Moroccan population, especially young adults whose level of knowledge was low. Thus, with the aim of adopting and ensuring a positive healthy lifestyle, and preventing themselves and their families from developing CVRFs such as hypertension, obesity, hypercholesterolemia, and eventually CVDs.

Limitations of the study

This study has certain limitations that must be acknowledged. It was a cross-sectional study based on a hard-copy questionnaire. This may represent a limitation, as papers are more likely to be damaged or lost, given that a number of copies were not completed. However, this problem was overcome by the large sample size, which represents the robustness of the study.

## Conclusions

The moderate knowledge regarding CVD among the general population of Morocco is a call for action to the necessity of adopting adequate educational programs to raise awareness regarding CVDs in Morocco. The present study emphasizes that high awareness regarding CVDs will lead to adequate behaviors to prevent the onset of preventable diseases especially among young adults. In addition, the implementation of massive and cost-effective health education is essential to minimize cardiovascular morbidity and mortality in Morocco.
